# Case Report: COVID-19 and Chagas Disease in Two Coinfected Patients

**DOI:** 10.4269/ajtmh.20-1185

**Published:** 2020-10-06

**Authors:** Ricardo Wesley Alberca, Tatiana Mina Yendo, Yasmim Álefe Leuzzi Ramos, Iara Grigoletto Fernandes, Luana de Mendonça Oliveira, Franciane Mouradian Emidio Teixeira, Danielle Rosa Beserra, Emily Araujo de Oliveira, Sarah Cristina Gozzi-Silva, Milena Mary de Souza Andrade, Anna Cláudia Calvielli Castelo Branco, Anna Julia Pietrobon, Nátalli Zanete Pereira, Cyro Alves de Brito, Raquel Leão Orfali, Valéria Aoki, Alberto José da Silva Duarte, Gil Benard, Maria Notomi Sato

**Affiliations:** 1 Departamento de Dermatologia, Faculdade de Medicina FMUSP, Laboratorio de Dermatologia e Imunodeficiencias (LIM-56), Universidade de Sao Paulo, Sao Paulo, Brazil;; 2 Institute of Biomedical Sciences, University of São Paulo, São Paulo, Brazil;; 3 Technical Division of Medical Biology, Adolfo Lutz Institute, Immunology Center, São Paulo, Brazil

## Abstract

American trypanosomiasis, also named Chagas disease (CD), is an anthropozoonosis caused by the protozoan parasite *Trypanosoma cruzi*. The disease affects millions of people worldwide, leading yearly to approximately 50,000 deaths. COVID-19, generated by SARS-CoV-2, can lead to lymphopenia and death. We hereby describe the first report of two patients with CD and COVID-19 coinfection, from hospitalization until patients’ death.

## INTRODUCTION

Chagas disease (CD) is a vector-borne disease transmitted mainly by the bloodsucking bug from subfamily Triatominae.^1^ Chagas disease is named after Carlos Chagas, a Brazilian researcher who described the life cycle of the parasite *Trypanosoma cruzi*. The disease was first described in Latin America,^1^ but currently it is a neglected health problem, affecting approximately 7 million people worldwide.^2^

Chagas disease presents two distinct clinical phases: an acute one, associated with a strong type 1 immune response,^3^ and a chronic phase, which may endure for the rest of the patients’ life.^3^ The chronic phase may evolve to different degrees of severity in 30% of the cases, leading to dilated cardiomyopathy, arrhythmia, cardioembolism, heart failure, and death.^3,4^ Systemic arterial hypertension (SAH) and dyslipidemia are also common features among elderly CD patients.^5^

A *Trypanosoma cruzi*–infected person can also progress to an indeterminate form, in which the patient has no symptoms or signs of the disease, presenting no alteration in gastrointestinal and myocardial functions.^6^ Nevertheless, coinfections can lead to CD reactivation and influence the overall prognoses of the patient.^7,8^

COVID-19 is a disease caused by SARS-CoV-2 infection that leads to high rates of respiratory illness and death.^9^ Several risk factors have been investigated, such as obesity, type 2 diabetes mellitus, and SAH.^10^

SARS-CoV-2 infection mainly affects the respiratory tract^11^ but recently has been associated with significant multiple organ dysfunction syndrome.^12^ One of the most common critical complications during COVID-19 is myocardial injury,^13,14^ especially in patients with a higher inflammatory profile.^13^

As the pandemic is still ongoing, it is impossible to precise the impact of SARS-CoV-2 infection in the overall population, but to the moment, COVID-19 has caused almost 1 million deaths worldwide. Although CD affects millions, to the moment, there is no report of CD and COVID-19 coinfection in the literature.

We analyzed two CD-infected patients (aged 69 and 74 years), with confirmed SARS-CoV-2 infection by nasopharyngeal detection of SARS-CoV-2 RNA, using reverse transcriptase–PCR and negative results for influenza and respiratory syncytial virus. The patients were hospitalized in a special ward for COVID-19 patients at the university hospital (Hospital das Clinicas, Faculdade de Medicina da Universidade de São Paulo[HCFMUSP]) with approval for the usage of data from patients. We hereby describe laboratory data from day one until both patients passed away. The study was approved by the local Ethics Committee (HCFMUSP no. 30800520.7.0000.0068-2020) and was carried out in conformity with the 2013 revision of the Declaration of Helsinki.

## CASE PRESENTATIONS

**Figure 1.**
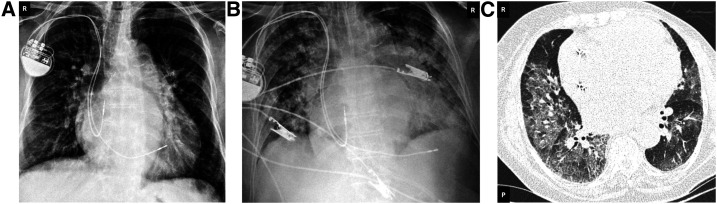
Chest X-ray and thoracic computerized tomography scan of patient #1. (**A**) X-ray from 2015 pre–COVID-19 hospitalization showing the marked cardiomegaly, (**B**) X-ray from June 2020, and (**C**) tomography from June 2020 during COVID-19 hospitalization showing COVID-19 ground-glass opacity.

The first patient was a woman, aged 74 years, who had COVID-19–associated symptoms on May 26, tested positive for SARS-CoV-2 on May 30, and hospitalized. She had a past diagnosis of CD, with chronic CD cardiomyopathy (Figure 1A) and a pacemaker since 2013 (Figure 1A) for a total atrioventricular block, and, on admission, atrial fibrillation with dilatation of the pulmonary artery trunk (43 mm). Additional past clinical history included a stroke in 2010 and a unilateral mastectomy of the left side due to breast cancer in 2011. The patient’s regular medications were hydralazine, atenolol, losartan, omeprazole, amiodarone, atorvastatin, and isosorbide. On admission, the patient presented dyspnea but not cough, fever, or myalgia. Chest radiography revealed pulmonary ground-glass opacities affecting 50% of the lungs (Figure 1B and C). The level of N-terminal pro–b-type natriuretic peptide (NT-proBNP) was 1,319 pg/mL (reference value of < 125 pg/mL), but D-dimer was below detection, but peaked in 1738 ng/mL in fibrinogen equivalent units (FEUs) on the 10th day at the hospital. Total bilirubin and fractions as well as total protein levels and fractions (albumin and globulin) were within reference levels. No alteration in the levels in serum alanine transaminase or aspartate transaminase was detected during hospitalization. As part of the COVID treatment protocol, she received ceftriaxone from June 1 to 6, methylprednisolone 40 mg on June 7, azithromycin from June 1 to 2, and warfarin on June 7.

**Figure 2.**
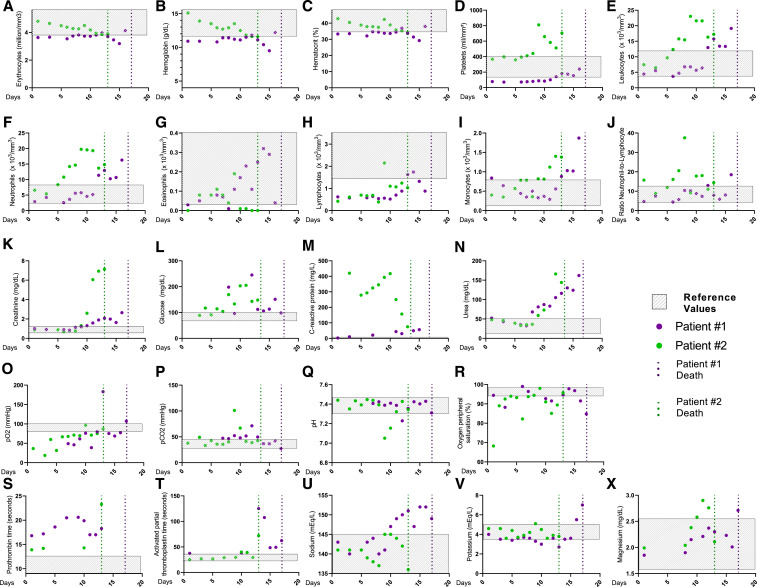
Daily clinical features of patients. Blood levels of (**A**) erythrocytes, (**B**) hemoglobin, (**C**) hematocrit, (**D**) platelets, (**E**) leukocytes, (**F**) neutrophils, (**G**) eosinophils, (**H**) lymphocytes, (**I**) monocytes, (**J**) neutrophil-to-lymphocyte ratio, (**K**) creatinine, (**L**) glucose, (**M**) C-reactive protein, (**N**) urea, (**O**) pO_2_, (**P**) pCO_2_, (**Q**) pH, (**R**) oxygen peripheral saturation, (**S**) prothrombin time, (**T**) activated partial thromboplastin time, (**U**) sodium, (**V**) potassium, and (**X**) magnesium. Gray box represents reference values; dot line represents the date the patients passed away.

Patient #1 showed normal levels of red blood count, hemoglobin, and hematocrit, but a platelet count reduction since day 1 of hospitalization (Figure 2A–D). White blood counts were normal in the first week, with an increase in neutrophils and monocytes by the end of the second week of hospitalization (Figure 2E–J). The neutrophil-to-lymphocyte ratio remained at normal levels during hospitalization due to the reduced lymphocyte cell count, a common feature in COVID-19 (Figure 2J).^15^

There was also a sustained elevation of creatinine levels, C-reactive protein, and urea (Figure 2K, M, and N). On day 8, the patient developed blood glucose alterations, probably caused by SARS-CoV-2 infection, as previously proposed (Figure 2L).^16^

Other findings included low pO_2_, but normal levels of pCO_2_ and pH, with a variable oxygen peripheral saturation (Figure 2O–R). High prothrombin time during COVID-19 was also verified (Figure 2Q). From day 10 onward, the patient presented altered sodium, potassium, and magnesium serum levels (Figure 2U–X). The patient’s clinical situation rapidly deteriorated, evolving into SARS. The patient was transferred to the ICU, and despite all efforts, the patient passed away on day 17 (June 17) because of refractory circulatory shock.

The second patient was a man, aged 69 years, weighing 62 kg, who had COVID-19–associated symptoms on May 7. He sought the hospital 2 weeks later, and tested positive on May 22 and hospitalized. He was a heavy smoker and has been followed at the HCFMUSP for indeterminate CD. Chest radiography revealed pulmonary ground-glass opacities in 50% of the parenchyma. On admission, the level of NT-proBNP was 458 pg/mL and of D-dimer was 88,868 ng/mL FEUs. Blood troponin T levels were within the normal range during admission (< 0.014 ng/mL), but increased to 0.193 ng/mL on the ninth day. Total bilirubin and fractions as well as total protein levels and fractions (albumin and globulin) were within reference levels. No alteration in the levels in serum alanine transaminase or aspartate transaminase was detected during hospitalization. During hospitalization, he received azithromycin and ceftriaxone from May 22 to 27, enoxaparin, methylprednisolone 30 mg on May 27, piperacillin/tazobactam from May 27 to 31, vancomycin from May 31 until death, and meropenem on May 31.

Patient #2 was admitted already with respiratory distress and immediately transferred to the ICU. Laboratory findings showed normal levels of erythrocytes and hemoglobin, and high levels of platelet since day 1 (Figure 2A–D). High counts on leukocytes, especially on neutrophils, were seen since day 5, with the maintenance of low lymphocyte counts (Figure 2E–J). Creatinine and urea levels were stable, with an abrupt spike on day 10 (Figure 2K and N). Interestingly, C-reactive protein levels were constantly high, and pO_2_ levels were regularly low, with pCO_2_ and pH levels under normal levels (Figure 2M and O–Q), but oxygen saturation was regularly below reference values (Figure 2R). Prothrombin time and activated partial prothrombin time increased on day 13 (Figure 2S and T). Sodium and potassium serum levels were always normal during hospitalization time, but the magnesium levels increased rapidly in the last couple of days in the hospital (Figure 2U–X). On day 8, there was an increase in the glucose serum levels, probably related to the SARS-CoV-2 infection (Figure 2L), as previously proposed.^16^

The patient progressed with pulmonary thromboembolism and SARS, followed by hypotension and cardiac arrest. Despite all efforts, the patient passed away on day 13 (June 3).

## DISCUSSION

We hereby describe two patients with CD and SARS-CoV-2 coinfection. Both patients were hospitalized in a reference center for COVID-19 treatment in the metropolitan region of São Paulo, a city in the Southeast of Brazil. The southeast region comprises less than 5% of the CD cases in Brazil. We hypothesize that CD and COVID-19 coinfection may be an important noninvestigated cause of death in regions with a higher incidence of CD.^17^

Chagas disease can manifest as a severe, life-threatening opportunistic infection in patients with immune deficiencies such as HIV.^18^ COVID-19 may lead to lymphopenia, which could curb the anti–*T. cruzi* immune response, similar to what is seen in HIV patients.^18^ Elderly CD patients are also prone to develop SAH and dyslipidemia,^5^ therefore being at higher risk group for COVID-19.^11,19^

Some patients with COVID-19 develop severe disease characterized by respiratory and systemic syndromes.^20^ Chronic underdiagnosed diseases, such as indeterminate form CD, and other coinfections,^21–23^ may alter the disease course and represent a significant risk factor for poor COVID-19 outcomes.

Nevertheless, advanced age in these two case reports could be an additional risk factor, reducing the immune response against CD and COVID-19.^5,24^ These may be due to SARS-CoV-2 cell entry receptor expression, angiotensin-converting enzyme-2, immunosenescence, or a larger number of medical comorbidities.^25^

This report highlights the first report of CD and COVID-19 coinfection. Patients presented a rapid disease progression, and despite all efforts of the medical team, both patients died. We believe that CD may be an important and underrated risk factor for developing severe COVID-19, especially those with chronic CD with cardiomyopathy that may be prone to have poor outcomes, especially in endemic areas with underreported CD infection and/or underreported SARS-CoV-2 infection.
